# Development of monoclonal antibodies against *P. gingivalis* Mfa1 and their protective capacity in an experimental periodontitis model

**DOI:** 10.1128/msphere.00721-24

**Published:** 2024-12-19

**Authors:** Mingya Cao, Siyu Wang, Shengke Zhou, Min Yan, Yu Zou, Yuan Cui, Xinyu Lou, Yichang Gao, Ying Chen, Zijing Han, Yi Qian, Jingying Chen, Xia Li

**Affiliations:** 1 Joint National Laboratory for Antibody Drug Engineering, School of Medicine, Henan University, Kaifeng, China; 2 The First Affiliated Hospital of Henan University, Henan University, Kaifeng, China; University of Galway, Galway, Ireland

**Keywords:** periodontitis, *P. gingivalis*, monoclonal antibodies, fimbriae, Mfa1

## Abstract

*Porphyromonas gingivalis* (*P. gingivalis*), a gram-negative, black-pigmented anaerobe, is a major etiological agent and a leading cause of periodontitis. Fimbriae protein Mfa1 is a key virulence factor of *P. gingivalis* and plays a crucial role in bacterial adhesion, colonization, biofilm formation, and persistent inflammation, making it a promising therapeutic target. However, the role of anti-Mfa1 antibodies and the underlying protective mechanisms remain largely unexplored. Here, we developed and characterized the monoclonal antibodies (mAbs) targeting the Mfa1 protein of *P. gingivalis*. Function analysis showed that anti-Mfa1 mAbs mediated bacterial agglutination and inhibited *P. gingivalis* adhesion to saliva-coated hydroxyapatite and host cells. Notably, anti-Mfa1 mAbs significantly reduced bacterial burden and alveolar bone loss in a *P. gingivalis*-induced experimental periodontitis model. These results show that anti-Mfa1 mAbs can be beneficial in alleviating *P. gingivalis* infections, and provide important insights for the development of adequate adjuvant treatment regimens for Mfa1-targeted therapeutics.

**IMPORTANCE:**

Fimbriae (pili) play an important role in bacterial adhesion, invasion of host cells and tissues, and formation of biofilms. Studies have shown that two types of fimbriae of *Porphyromonas gingivalis*, FimA and Mfa1, are important for colonization and infection through their binding to host tissues and other bacteria. While anti-FimA antibodies have been shown to improve periodontitis, the effect of anti-Mfa1 antibodies on *P. gingivalis* infection and periodontitis was previously unknown. In this study, we report for the first time that anti-Mfa1 monoclonal antibodies can reduce *P. gingivalis* infection and improve periodontitis. These findings suggest that Mfa1 represents a promising therapeutic target, and the development of anti-Mfa1 mAbs holds a potential as essential diagnostic and adjunctive therapeutic tools for managing *P. gingivalis*-related diseases.

## INTRODUCTION

*Porphyromonas gingivalis* is a black-pigmented, gram-negative anaerobic bacterium that thrives in the subgingival crevices around teeth, disrupting host immunity and dysregulating the periodontal microbial ecology, leading to persistent inflammation and chronic periodontal disease (1). Furthermore, *P. gingivalis* is thought to contribute to the development and progression of a variety of systemic diseases, including rheumatoid arthritis, cardiovascular diseases, diabetes, pregnancy complications, Alzheimer’s disease, and numerous types of cancer (2–4). The virulence of *P. gingivalis* is determined by a variety of its cell surface molecules and extracellular toxins, such as fimbriae, lipopolysaccharide, hemagglutinin, and gingipains (Arg- and Lys-specific proteases) (5, 6). These virulence factors facilitate *P. gingivalis* adhesion to gingival tissues, promote biofilm formation and host cell invasion, leading to oral microbial dysbiosis and the development of chronic inflammation, ultimately resulting in the onset and progression of periodontitis.

Fimbriae (pili) are proteinaceous, filamentous appendages that protrude from the bacterial cell surface, one of the major virulence factors of *P. gingivalis*. Usually, two distinct types of fimbriae (Fim fimbriae and Mfa fimbriae) are expressed in the *P. gingivalis*, each fimbria is composed mainly of FimA and Mfa1 protein polymers encoded by the gene of *fimA* and *mfa1* in each gene clusters, respectively (7, 8). They play crucial roles in biofilm formation, auto-aggregation, co-aggregation with oral bacteria, adhesion to host molecules, and host cell invasion (6, 8). Mutations or inactivation of FimA protein diminishes the adhesive properties of *P. gingivalis*, thus attenuating alveolar bone destruction in periodontitis (9). Mfa1 is a pivotal structural component of the Mfa fimbriae, with Mfa2-5 proteins aiding in its assembly, stability, and adhesion functions (7, 10). This protein is crucial not only for the development of *P. gingivalis* biofilms on *Streptococcus gordonii* surfaces (11) but also for its survival in host dendritic cells by binding to DC-SIGN, thereby evading autophagy and lysosomal fusion (12, 13). Mfa1 fimbriae have also been shown to elicit a proinflammatory response in monocytes and macrophages, upregulating the expression of IL-1β and IL-6 cytokines via TLR2 and CD14 signaling pathways (13, 14). Although the role of Mfa1 in *P. gingivalis* physiology and pathogenicity is not fully understood, it is evident that Mfa1 plays a significant role in the bacterium’s adhesion properties. An Mfa1 deletion mutant (MPG67) demonstrated significantly attenuated alveolar bone loss in a rat periodontitis model compared to both wild-type *P. gingivalis* and a FimA mutant strain (9). The mutant also displayed alterations in biofilm structure and maturation, suggesting that Mfa1 contributes to the prevention of auto-aggregation and biofilm formation (15, 16). These findings suggest that Mfa1 is an important mediator of *P. gingivalis* adhesion, colonization, and pathogenesis, making it a promising target for therapeutic intervention.

To date, the development of mAbs targeting Mfa1 and their role *in vivo* have not been reported. In this study, we expressed and purified the Mfa1 protein, generated a series of anti-Mfa1 mAbs by immunizing mice and employing hybridoma technology, and subsequently assessed their affinity and specificity. We demonstrate that these mAbs increase aggregation and reduce the adhesion function of *P. gingivalis*. Through *in vivo* experiments, we confirmed that anti-Mfa1 mAbs can significantly reduce bacterial load and alveolar bone resorption in a rat periodontitis model induced by *P. gingivalis*. Our results suggest that specific anti-Mfa1 mAbs may be a potential component of diagnostic tools and antibody-based adjuvant therapies for *P. gingivalis* infection.

## RESULTS

### Expression of Mfa1 protein and generation of anti-Mfa1 mAbs

**Fig 1:**
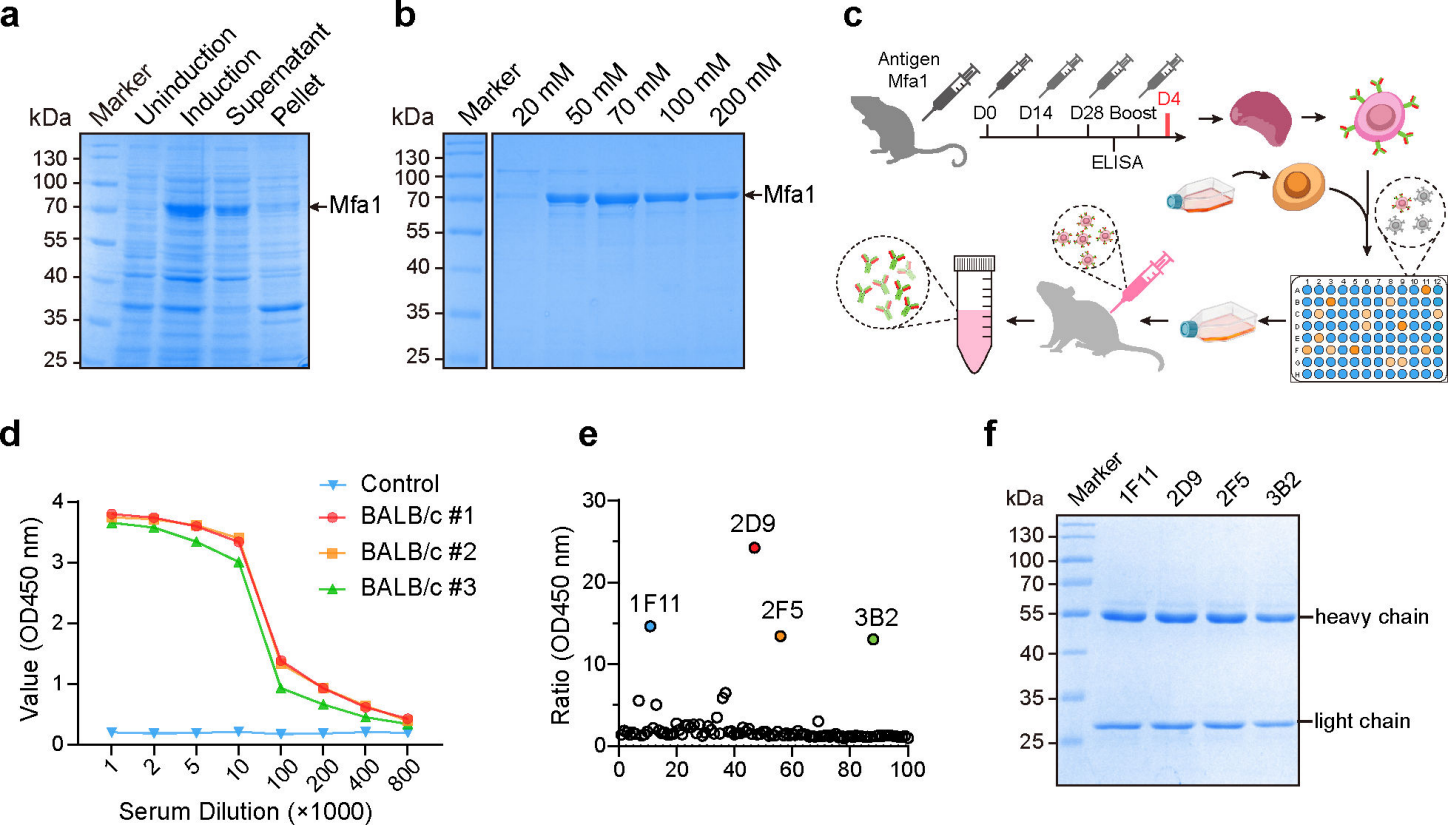
Expression of Mfa1 protein and generation of mAbs against *P. gingivalis* Mfa1. (**a and b**) Expression and purification of recombinant Mfa1 protein. SDS-PAGE analysis of the pET28a-Mfa1 in *E. coli* BL21(DE3) cells. Lane M, protein molecular marker; lane 1, total protein supernatant before induction; lane 2, supernatant sample before purification; lane 3, flowthrough sample after purification; lane 4–8, purified Mfa1 was eluted with 20, 50, 70, 100, and 200 mM imidazole in equilibration buffer (pH 8.0). (**c**) Schematic view of immunization, hybridoma generation, and screening for obtention of anti-Mfa1 mAbs. (**d**) Determination of serum antibody titers by ELISA. Serum antibodies were detected using recombinant protein Mfa1-coated ELISA plates, and serum antibody titers from all three immunized mice reached 1:200,000. (**e**) More than 100 hybridoma clone culture supernatants were collected and screened by ELISA to finally obtain four different hybridoma cell lines capable of secreting Mfa1 monoclonal antibodies. (**f**) SDS-PAGE analysis of four anti-Mfa1 mAbs purified from mouse ascites via protein G affinity purification.

In order to study and develop more effective monoclonal antibodies against Mfa1, the Mfa1 gene was cloned into the pET-28a vector and expressed in *Escherichia coli* BL21 (DE3) (Fig. 1a). The His-tagged Mfa1 protein was successfully purified by nickel affinity chromatography using a HisTrap FF column (Fig. 1b). SDS-PAGE results showed a major protein band at the expected 70 kDa. These preparations were used as antigens for antibody production and standards for immunoassays. Three BALB/c mice were immunized with His-tagged Mfa1 recombinant protein (Fig. 1c). After three immunizations, enzyme-linked immunosorbent assay (ELISA) results showed that the mice produced antibodies with high binding potency more than 400,000-fold (Fig. 1d). Anti-Mfa1 hybridomas were generated by fusing immortalized myeloma cell fusion with the splenocytes from BALB/c mice with the highest serum titer. We screened more than 100 hybridoma colonies and identified four secreting hybridomas (1F11, 2D9, 2F5, and 3B2) that secreted antibodies with strong binding to intact Mfa1, using ELISA as the screening method (Fig. 1e). Culture of the four positive hybridoma cell lines was expanded, and they were injected into mice. Subsequently, the four anti-Mfa1 mAbs were purified from mouse ascites by Protein A affinity chromatography and were analyzed by SDS-PAGE to confirm their identity and purity (Fig. 1f; Fig. S1a). ELISA isotyping analysis revealed that mAbs 1F11, 2F5, and 3B2 were predominantly of the IgG1 isotype, while 2D9 had an IgG2b isotype. All four mAbs had kappa light chains (Fig. S1b).

### Potency and affinity determination of anti-Mfa1 mAbs

**Fig 2:**
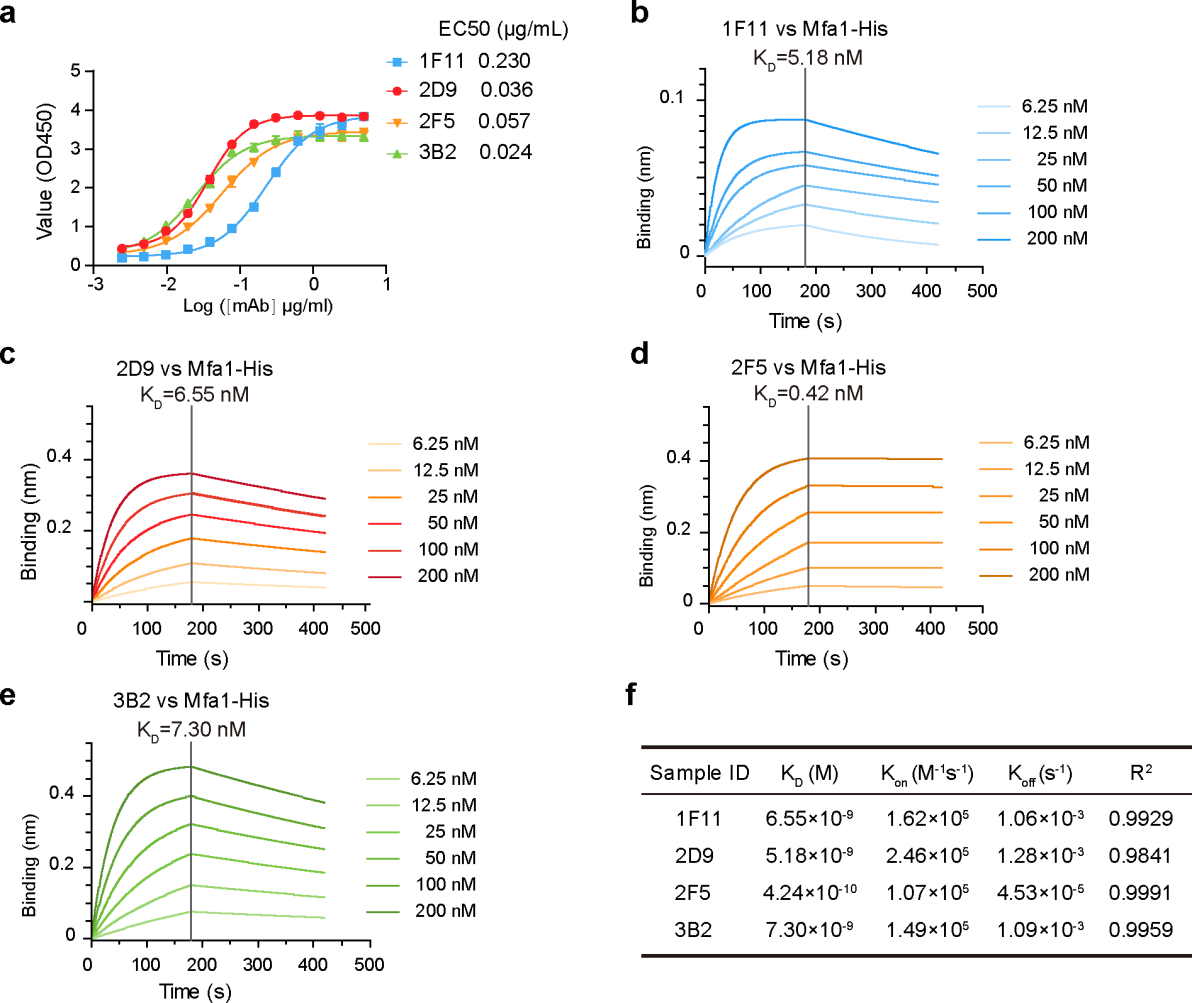
Analysis of mAbs binding to Mfa1 by ELISA and BLI. (**a**) Binding as reflected by absorbance at 450 is shown on the *y* axis for the mAbs concentrations shown on the *x* axis for each mAb. Results are representative of three independent experiments. The numerical EC50 for each mAb is indicated at the right of the panel depicting the binding curves of all mAbs. (**b–e**) Affinity-binding curves of anti-mAbs were measured by BLI. BLI analysis was used on the Octet R8 system to determine the association and dissociation rates by immobilizing the monoclonal antibody on an AMC-coated optical sensor. The AMC sensors were incubated with purified monoclonal antibody at set intervals to allow binding. The sensor is then moved into an antibody-free solution and allowed to dissociate at intervals of time. Curve fitting using a 1:1 interaction model allows the measurement of the affinity constant (Kd) of each nanobody. (**f**) Curve fitting using a 1:1 interaction model allows for the affinity constant (Kd) to be measured for each anti-Mfa1 mAbs.

To evaluate the Mfa1-binding capacity of the four mAbs, we performed ELISA and biolayer interferometry (BLI) assays. ELISA data showed that mAb 3B2 had the highest binding capacity (50% effective concentration [EC50], 0.024 µg/mL), followed by 2D9 (EC50, 0.036 µg/mL) and 2F5 (EC50, 0.057 µg/mL). mAb-1F11 showed a weaker binding capacity with an EC50 value of 0.230 µg/mL (Fig. 2a). BLI data revealed that all four mAbs displayed a high-affinity binding to Mfa1 with Kd values in the nanomolar range: 1F11 (5.18 nM), 2D9 (6.55 nM), 2F5 (0.42 nM), and 3B2 (7.30 nM) (Fig. 2b through e). Notably, 2F5 exhibited a particularly tight binding with a Kd value in the sub-nanomolar range, indicating its ability to form a very stable complex with Mfa1 (Fig. 2d). In addition, 1F11 had a lower binding upper limit compared to the other three antibodies, which is consistent with the results of ELISA (Fig. 2a and b). The comparative analyses revealed that mAbs 2D9, 2F5, and 3B2 had a higher affinity for Mfa1 than mAb 1F11 (Fig. 2f). As a result, these three mAbs, 2D9, 2F5, and 3B2, were selected for further characterization and research to elucidate their potential therapeutic effects in *P. gingivalis*-induced periodontitis.

### Characterization of the specificity of anti-Mfa1 mAbs

**Fig 3:**
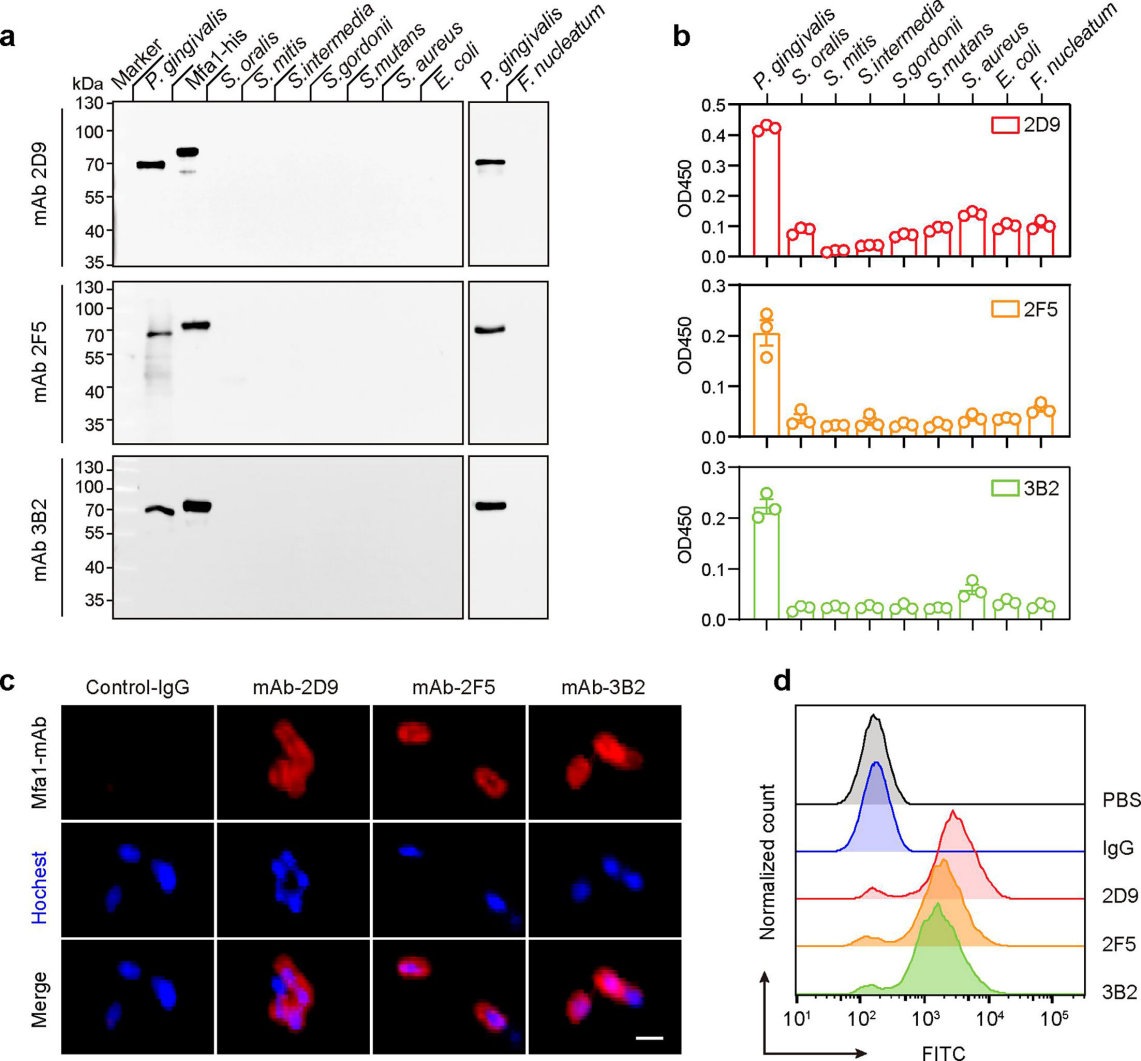
Specificity and cross-reactivity analysis of anti-Mfa1 mAbs. (**a**) Validation of monoclonal antibodies by Western blot analysis. Total cell lysates (20 µg per lane) prepared from *P. gingivalis* and other bacteria were analyzed by Western blot using anti-Mfa1 mAbs; the recombinant Mfa1 protein (2 µg) was as a positive control. (**b**) Detection of cross-reactivity of anti-Mfa1 monoclonal antibodies by ELISA. (**c**) Visualization of mAbs binding to *P. gingivalis* cells. Nuclei DNA was labeled with Hochest, while mAb-coated bacteria were stained with 546-conjugated anti-mouse IgG antibody (scale bar: 2 µm). (**d**) Flow cytometric analysis of binding to *P. gingivalis* using anti-Mfa1 mAbs and non-specific IgG (blue). *P. gingivalis* was incubated with anti-Mfa1 mAbs, reacted with 488-conjugated anti-mouse IgG, and detected by flow cytometry.

To assess the specificity of mAbs 2D9, 2F5, and 3B2, ELISA assays were performed with FimA (an alternative fimbrial protein) and RgpB (an Arg-specific protease). The results showed specific binding of the mAbs to Mfa1, with no binding to the other two proteins (Fig. S1c). To confirm the specificity of mAbs 2D9, 2F5, and 3B2, we conducted a Western blot assay using total bacterial proteins of *P. gingivalis* and other bacterial organisms. Our results showed that these mAbs only interact with the Mfa1 of *P. gingivalis* and the recombinant Mfa1 protein. None of them cross-reacted with other bacterial strains, such as *Streptococcus oralis*, *Streptococcus intermedia*, *Streptococcus mitis*, *S. gordonii*, *Streptococcus mutans*, *Staphylococcus aureus*, *E. coli*, and *Fusobacterium nucleatum* (Fig. 3a). To further analyze the specificity of these mAbs binding to Mfa1 under native conditions, we performed an ELISA assay with bacteria coated on the plate. The results were consistent with the Western blot findings, showing that the anti-Mfa1 mAbs have strong binding activity to *P. gingivalis* and have no cross-reactivity with other bacteria (Fig. 3b; Fig. S1d). Because Mfa1 is the surface-exposed fimbriae protein of *P. gingivalis* (6), we further assessed the binding of mAbs to *P. gingivalis* whole bacteria. For this purpose, indirect immunofluorescence assay and bacterial flow cytometric assay were performed to confirm the surface-bound interaction. The results showed that Mfa1 mAbs readily bound to *P. gingivalis*, while the non-specific IgG displayed no detectable surface binding (Fig. 3c and d). Together, these results confirmed that our mAbs exhibit a high-affinity and specificity binding to *P. gingivalis* Mfa1 protein and do not cross-react with other species.

### Anti-Mfa1 mAbs induce *P. gingivalis* auto-agglutination

**Fig 4:**
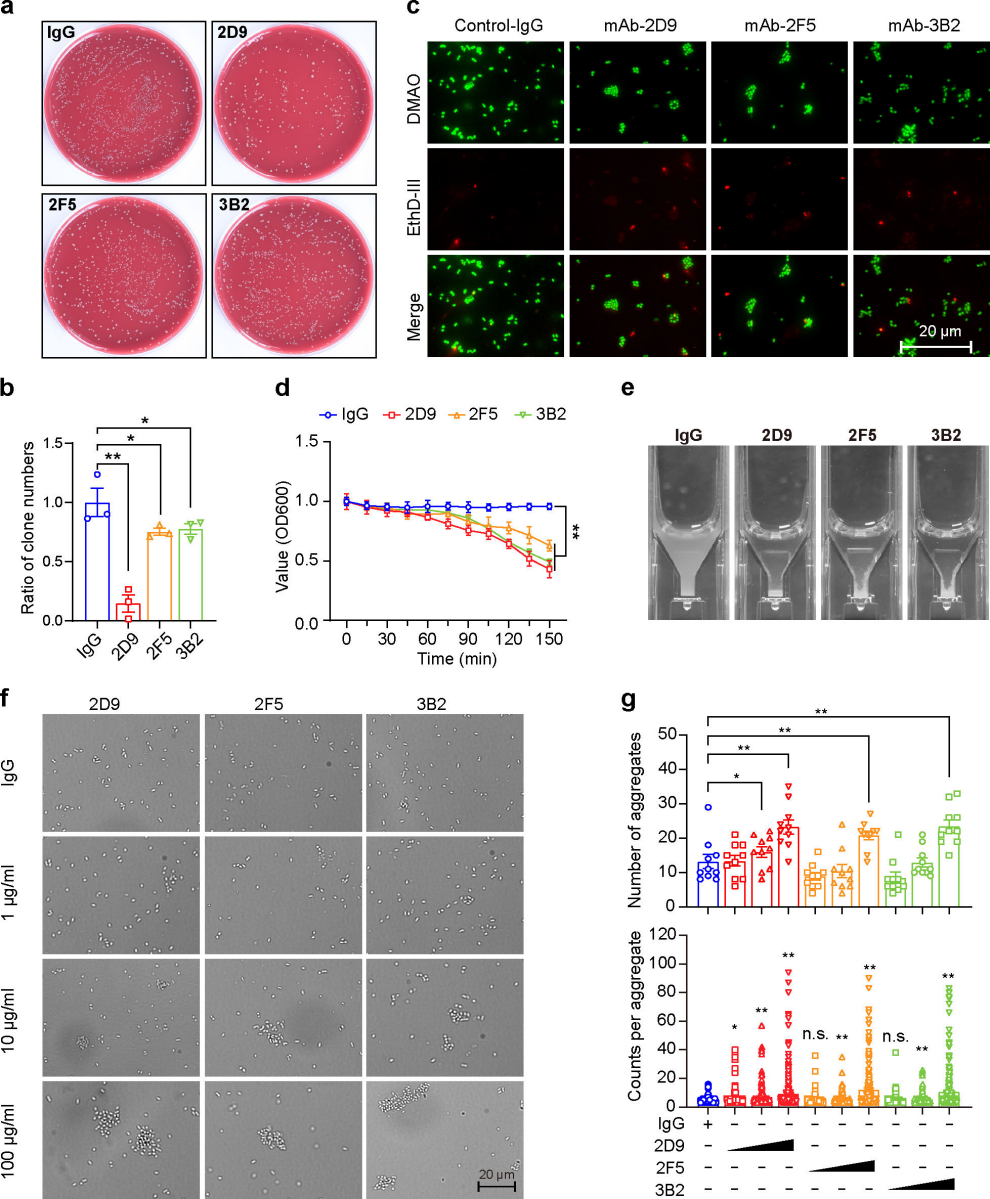
Anti-Mfa1 mAbs promote *P. gingivalis* agglutination. (**a**) Images of colony-forming assay of *P. gingivalis* after treatment with anti-Mfa1 mAbs and non-specific antibody. (**b**) Proportion of *P. gingivalis* inhibited after 2 h of incubation with anti-Mfa1 mAbs and non-specific IgG. (**c**) Images obtained from fluorescence microscopy of *P. gingivalis* after incubation with different anti-Mfa1 mAbs and non-specific IgG and briefly vortexing. (**d**) In a UV-clear cuvette, *P. gingivalis* was incubated with anti-Mfa1 mAb (100 µg/mL), and the absorbance at OD600 nm was continuously measured for 2.5 hours. (**e**) After incubation for 2.5 h, the cuvettes were photographed for visual inspection of turbidity. (**f**) A total of 2 × 10^8^ CFU of *P. gingivalis* were incubated with different purified anti-Mfa1 mAbs in PBS for 1 h. After gently vortexing, 10 µL of the mixture was dropped onto a glass slide, observed, and photographed under a microscope. Images are representative of the three independent experiments performed in duplicate for each mAb (scale bar: 20 µm). (**g**) More than 20 random area images were analyzed with a custom macro in ImageJ for the number of aggregates per image and the counts of bacteria in aggregates. Mean and standard error of the mean (s.e.m.) were calculated from the results of at least three independent experiments. Significant differences compared to the controls were determined using one-way ANOVA (*P < 0.05, **P < 0.01).

To evaluate the antibacterial activity of mAbs 2D9, 2F5, and 3B2, *P. gingivalis* was incubated with these mAbs or with non-specific IgG for 2 h at 37°C. Following briefly vortexing, the bacterial mixtures were plated on blood agar plates for enumeration of CFU. Our results revealed that *P. gingivalis* incubated with 2D9, 2F5, and 3B2 demonstrated a significant reduction in CFU compared to the IgG group (Fig.4a and b). Notably, larger colonies were observed more frequently in the mAbs-treated cultures. This finding prompts us to speculate that these mAbs may induce antibody-mediated bacterial agglutination, a phenomenon that could explain the higher frequency of larger colonies. To verify this hypothesis, we further used the LIVE/DEAD backlight bacterial viability kit to stain the treated bacteria, and used fluorescence microscopy and flow cytometry to determine bacterial aggregation and bacterial viability. The results indicated that the anti-Mfa1 mAbs induced aggregation of *P. gingivalis*, and vortexing for a brief period was insufficient to completely eliminate the aggregates (Fig. 4c). Furthermore, we observed that most of the anti-Mfa1 mAbs had little effect on *P. gingivalis* growth, with the exception of 2D9, which exhibited a mild growth inhibitory activity (Fig. S1e and f). This may be due to the greater aggregation of bacteria induced by 2D9, leading to a decrease in the rate of bacterial growth and colony formation.

Antibody-mediated bacterial agglutination is an important immune mechanism that helps prevent bacterial colonization and reduce infection (17). To further examine the role of bacterial agglutination in protection by anti-Mfa1 mAbs, a microbial agglutination assay was conducted. *P. gingivalis* was incubated with anti-Mfa1 mAbs and non-specific IgG at room temperature, and the absorbance at 600 nm was measured continuously. The results showed a significant decrease in absorbance values in a mixture containing 2D9, 2F5, and 3B2 compared to the non-specific IgG, with significant reduction at all time points starting from 75 min (Fig. 4d). Visualization of the cuvette showed a clear difference after 4 h of incubation (Fig. 4e). To determine if the decrease in turbidity was due to bacterial aggregation and precipitation, we visualized the resuspended aggregates through Axio ImagerA2 system and conducted a quantitative analysis through photographs of no fewer than 20 fields of view. Visualization of *P. gingivalis* treated with increasing mAbs concentrations showed a significant increase in bacterial aggregates (Fig. 4f). Furthermore, quantitative data showed that the number and size of aggregates were positively correlated with antibody concentration (Fig. 4g). These findings suggest that anti-Mfa1 mAbs induce agglutination of *P. gingivalis*, thereby potentially hindering its adhesion to saliva-coated hydroxyapatite (sHA) and host cells.

### Effect of anti-Mfa1 mAbs on *P. gingivalis* adhesion and biofilm formation

**Fig 5:**
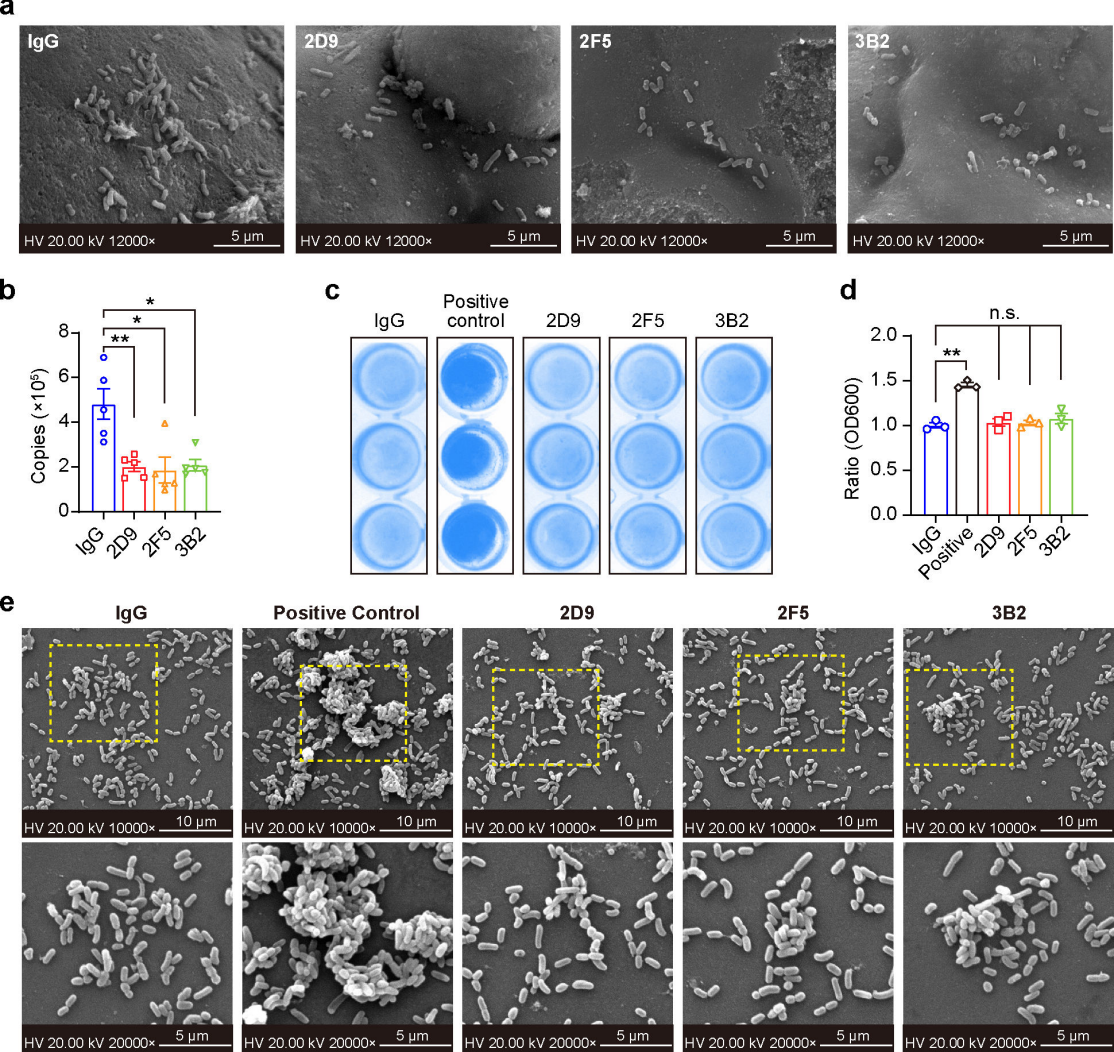
Anti-Mfa1 mAbs reduced the attachment of *P. gingivalis* to sHA. (**a**) The effect of anti-Mfa1 mAbs on *P. gingivalis* binding to sHA microspheres was observed by scanning electron microscopy. The image is representative of the triplicate experiment. (**b**) qPCR assay was used to evaluate the inhibitory effect of anti-Mfa1 mAbs on *P. gingivalis* binding sHA beads. (**c**) Representative pictures of biofilm formed in the presence of indicated mAbs after crystal violet staining. An anti-FimA monoclonal antibody that can increase biofilm is used as a positive control. (**d**) Biofilm biomass was assessed by absorbance at 600  nm. Mean and s.e.m. were calculated from the results of at least three independent experiments. (e) SEM micrographs of *P. gingivalis* exposed to the respective antibodies, including non-specific IgG, a FimA mAb as a positive control, and different anti-Mfa1 mAbs, for 48 h. Significant differences compared to the control IgG were determined using one-way ANOVA (*P < 0.05, **P < 0.01).

To investigate the effect of anti-Mfa1 mAbs on *P. gingivalis* initial adhesion, sHA beads were incubated with *P. gingivalis* treated with mAb 2D9, 2F5, 3B2, and non-specific IgG for 2 h. Scanning electron microscopy (SEM) results obtained after 4 h of co-incubation showed a decrease in the number of *P. gingivalis* cells in all three mAb-treated groups compared to the control group (Fig. 5a). Additionally, we evaluated adhesion to sHA by detecting adherent bacteria by *P. gingivalis* DNA copy number. Quantitative PCR (qPCR) results showed that *P. gingivalis* adhesion to sHA was significantly reduced after mAb incubation treatment, consistent with the SEM results (Fig. 5b). To further evaluate the effect of anti-Mfa1 mAbs on *P. gingivalis* pathogenesis, we examined the role of these antibodies in *P. gingivalis* homotypic biofilm formation by using the crystal violet method and SEM. Crystal violet staining revealed that the anti-Mfa1 mAbs did not significantly alter the amount of *P. gingivalis* biofilm formation compared to non-specific IgG and a positive control (a FimA monoclonal antibody) (Fig. 5c and d). SEM analysis of biofilms also supported these findings, again indicating that three anti-Mfa1 mAbs showed little impact on the development of *P. gingivalis* biofilm formation (Fig. 5e). These results demonstrate that mAb 2D9, 2F5, and 3B2 induce bacterial aggregation but do not affect the homotypic biofilm formation of *P. gingivalis*.

### Anti-Mfa1 mAbs inhibit *P. gingivalis* adhesion to epithelial cells

**Fig 6:**
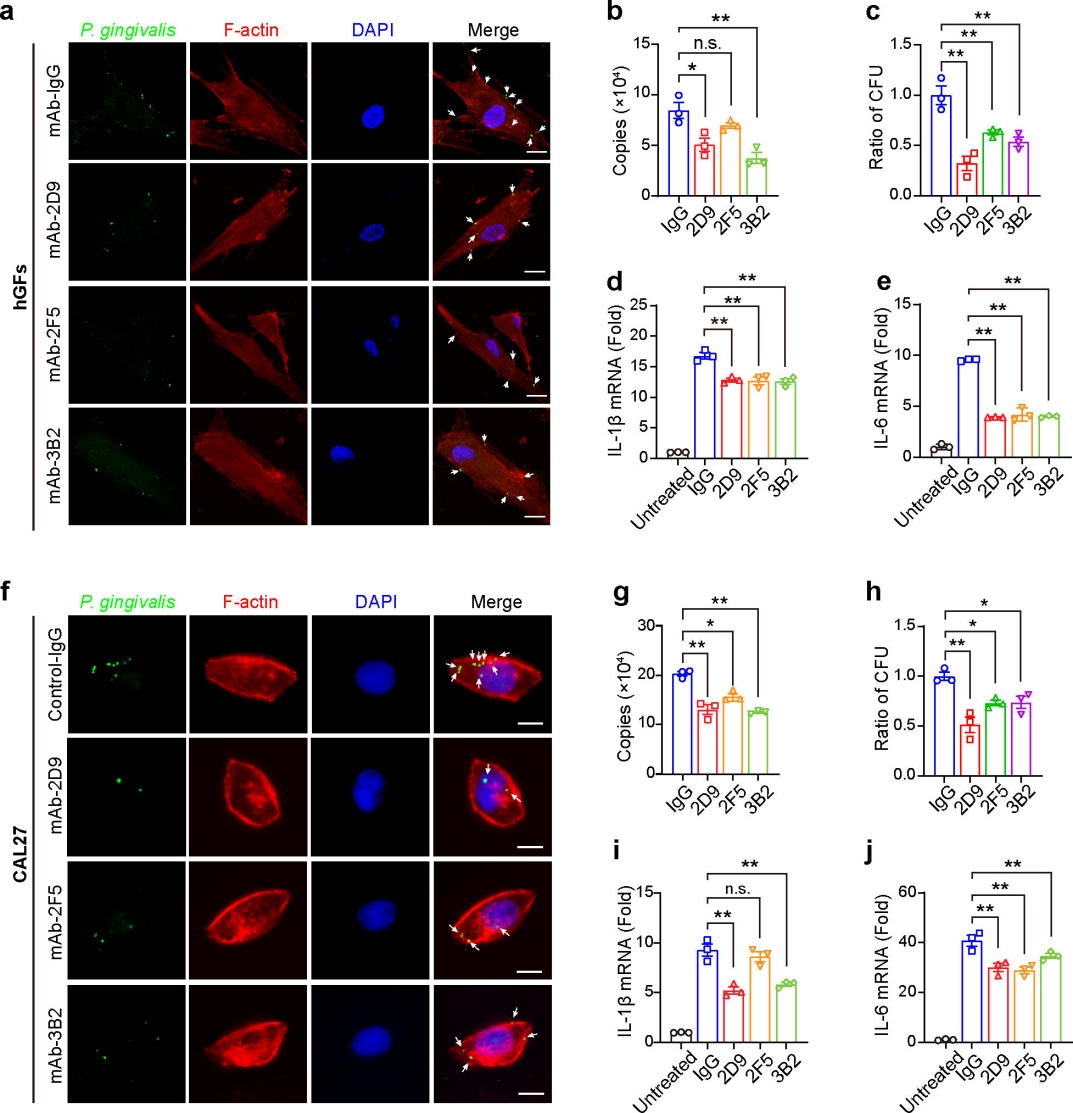
Anti-Mfa1 mAbs reduce the adhesion of *P. gingivalis* to host cells. (**a**) Immunofluorescence microscopy of the inhibitory effect of anti-Mfa1 mAbs on *P. gingivalis* binding hGFs. (**b**) Quantification of *P. gingivalis* adhered to hGFs after treatment with anti-Mfa1 mAbs by qPCR. (**c**) Antibiotic protection assay. Bars indicate the ratio of intracellular *P. gingivalis* recovered from hGF cells in different anti-Mfa1 mAbs treatment groups, as compared to the IgG control group. (**d and e**) Pro-inflammatory cytokines interleukin-1β (IL-1β) and interleukin-6 (IL-6) in hGFs infected with *P. gingivalis* (MOI = 10) for 24 h. (**f**) Immunofluorescence microscopy of the inhibitory effect of anti-Mfa1 mAbs on *P. gingivalis* binding hGFs. (**g**) Quantification of *P. gingivalis* adhered to CAL27 cells after treatment with anti-Mfa1 mAbs by qPCR. (**h**) Bars indicate the ratio of intracellular *P. gingivalis* recovered from hGFs cells in different anti-Mfa1 mAbs treatment groups, as compared to the IgG control group. (**i and j**) Relative mRNA expression of IL-1β and IL-6 in CAL27 infected with *P. gingivalis* (MOI = 10), treated by anti-Mfa1 mAbs for 24 h. Mean and s.e.m. were calculated from results of at least three independent experiments. *P < 0.05, **P < 0.01 as determined by one-way ANOVA.

Given the key role of *P. gingivalis* adhesion and invasion of host cells in the pathogenesis of periodontitis (4), we evaluated the effects of anti-Mfa1 mAbs on *P. gingivalis* adhesion and invasion in human gingival fibroblasts (hGFs). Using fluorescence microscopy and qPCR to assess *P. gingivalis* DNA in host cells, we found that mAbs 2D9, 2F5, and 3B2 significantly inhibited the adherence and invasion of fluorescein isothiocyanate (FITC)-labeled *P. gingivalis* to hGFs compared to the non-specific IgG control (Fig. 6a). The inhibitory effects of mAbs 2D9 and 3B2 on *P. gingivalis* adhesion and invasion were statistically significant, whereas mAb 2F5 showed a non-significant but similar trend (*P* = 0.08) (Fig. 6b). To further validate the observations from microscopic analysis and qPCR data, we performed antibiotic protection and CFU assays. The results confirmed that these anti-Mfa1 mAbs significantly inhibited the adhesion and invasion of hGFs by *P. gingivalis*, demonstrating the potential of these mAbs to prevent the attachment and entry of this pathogen into host cells (Fig. 6c). Human gingival fibroblasts have been identified as important players in the host response to *P. gingivalis*, participating in interactions with the bacteria and producing inflammatory cytokines such as IL-1β and IL-6, which have been linked to the pathogenesis of periodontitis (4). To further investigate the potential anti-inflammatory effects of anti-Mfa1 mAbs, we assessed the expression of these cytokines in hGFs upon treatment with *P. gingivalis* and anti-Mfa1 mAbs. The results revealed that hGFs treated with *P. gingivalis* in the presence of anti-Mfa1 mAbs 2D9, 2F5, and 3B2 had significantly reduced levels of IL-1β and IL-6 mRNA, compared to IgG control, suggesting that these antibodies may help mitigate the inflammatory responses associated with *P. gingivalis* infection (Fig. 6d and e).

*P. gingivalis* has been implicated in the development of oral squamous cell carcinoma (OSCC), and OSCC cell lines are frequently employed to investigate mechanisms of *P. gingivalis* infection (18). In this study, we investigated the effect of anti-Mfa1 mAbs on *P. gingivalis* infection of CAL27 cells. The results were similar to those obtained with hGFs, as mAbs 2D9, 2F5, and 3B2 significantly inhibited the adhesion and invasion of CAL27 cells by *P. gingivalis* strains compared to non-specific IgG treatment (Fig. 6f through h). Transcription levels of IL-1β and IL-6 mRNA were significantly reduced in CAL27 cells following treatment with anti-Mfa1-mAb (Fig. 6i and j) , although IL-1β secretion was not observed in the 2F5-treated group, which may be due to differences in inflammatory response profiles between CAL27 and HGF cells, as previously reported (19). Taken together, these results indicate that anti-Mfa1 mAbs 2D9, 2F5, and 3B2 effectively block the *P. gingivalis* adhesion and reduce the expression of inflammatory cytokines.

### mAb 2d9 inhibits *P. gingivalis* infection and alleviates periodontitis pathology

**Fig 7:**
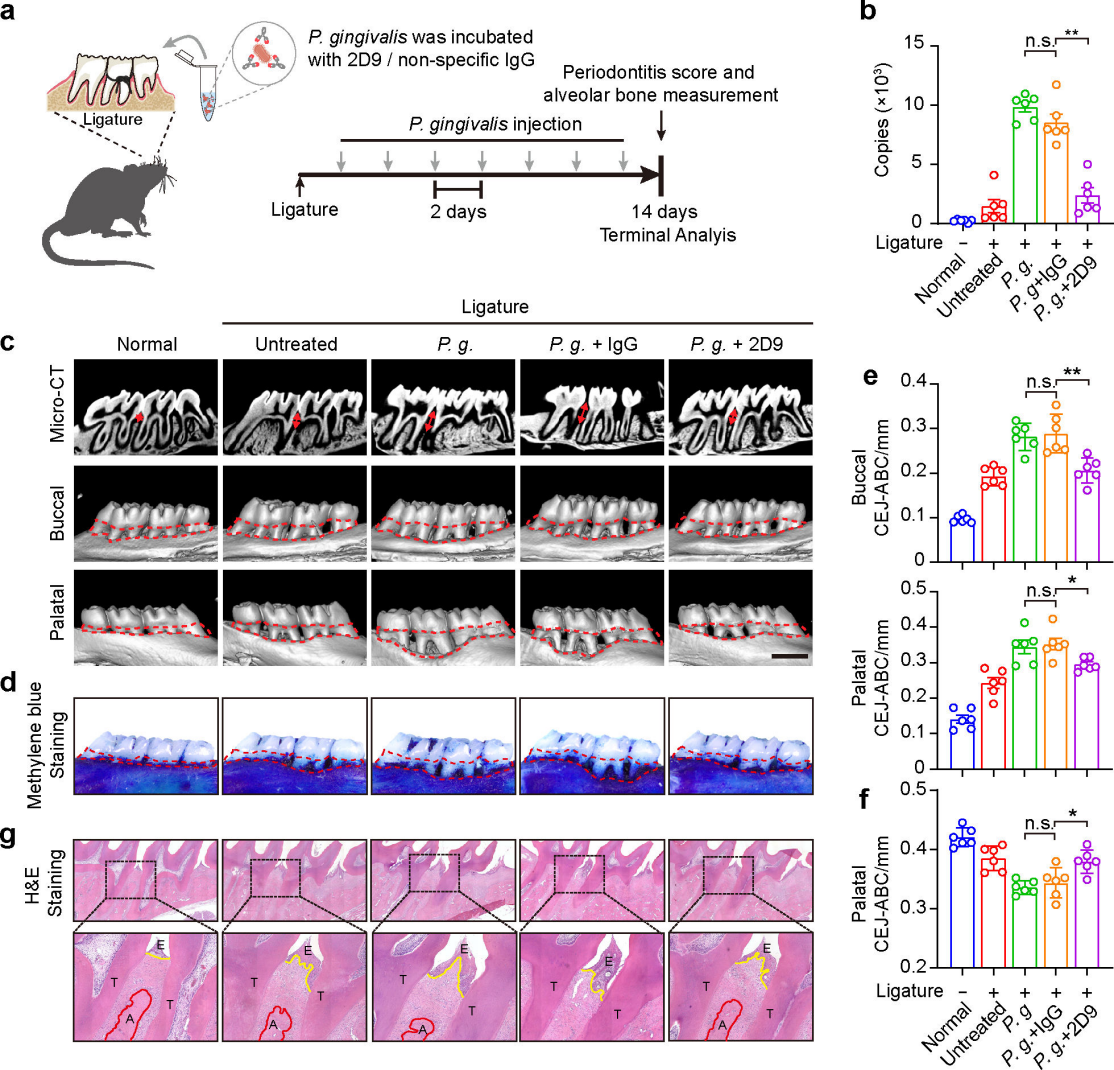
Anti-Mfa1 mAbs reduce *P. gingivalis* infection and improve experimental periodontitis symptoms. (**a**) Schematic workflow timeline of rat periodontitis model. (**b**) Quantification of *P. gingivalis* in rat oral cavity by qPCR. (**c**) Representative images of alveolar bone micro-CT analysis by different treatment groups. (**d**) Samples of the maxilla were collected and stained with methylene blue aqueous solution (1%) to distinguish bone from teeth. (**e**) Rat tissue sections were stained with H&E. (**f**) The average distance from the ABC to the CEJ on the buccal sides and the palatal sides of the maxillary second molar was measured. (**g**) Representative H&E staining images showing the alveolar bone resorption and periodontal inflammation of the periodontium from each group. Data are shown as means  ±  s.e.m. (*n* = 6); *P < 0.05, **P < 0.01 as determined by a one-way ANOVA test.

mAbs have emerged as a promising therapeutic strategy for treating bacterial infections due to their ability to target and neutralize specific pathogens with high precision. Previous studies have demonstrated that anti-FimA mAbs can effectively inhibit *P. gingivalis* adhesion and mitigate periodontal disease progression (20, 21). Here, we sought to investigate the therapeutic potential of anti-Mfa1 mAbs in a rat model of ligature-induced periodontitis with subsequent *P. gingivalis* infection. mAb 2D9 was chosen for *in vivo* testing because of its demonstrated high affinity, specificity, and antibacterial activity *in vitro*. As shown in Figure 7a, we developed a rat periodontitis model by ligating the second molars of Sprague-Dawley (SD) rats with cotton sutures and orally infecting *P. gingivalis* incubated in the presence or absence of 2D9, and the therapeutic effects were assessed by bacteria burden, alveolar bone atrophy, and hematoxylin and eosin (H&E) staining. Similar to the results observed *in vitro*, the *P. gingivalis* copy number was significantly increased in the *P. gingivalis* infected group and non-specific IgG group compared with the normal group and the ligatured untreated group. Pre-incubation with mAb 2D9 significantly reduced the copy number of *P. gingivalis* in rat oral cavity compared to *P. gingivalis* and IgG group (Fig. 7b). The micro-computed tomography (micro-CT) and methylene blue staining reflected alveolar bone loss by measuring the distance between the cement-enamel junction (CEJ) and alveolar bone crest (ABC) (red dotted line). Infection with *P. gingivalis* or IgG pre-incubated groups resulted in a substantial increase in alveolar bone loss. However, the mAb 2D9 group exhibited comparable levels of bone loss to the untreated group (Fig. 7c through f). Further histopathological studies by H&E staining observed that infection with *P. gingivalis* caused intense alveolar bone resorption and epithelial destruction. Rats infected with *P. gingivalis* pre-incubated with mAb 2D9 showed less bone loss than the *P. gingivalis* group and non-specific IgG group, with improvements in epithelial integrity, periodontal pocket depth, and periodontal ligament integrity (Fig. 7g). These results demonstrate that mAb 2D9 alleviated bone loss and improved periodontitis scores in the ligature-induced periodontitis model, which supported the potential of antibodies targeting Mfa1 as antibacterial therapeutic approaches.

## DISCUSSION

Targeting pathogenic bacteria’s virulence factors with mAbs is considered a promising strategy in the development of diagnostics and treatments for bacterial infections (22). Extensive research has been conducted on the use of antibacterial mAbs against various pathogens, including *S. aureus*, *Pseudomonas aeruginosa*, and *Acinetobacter baumannii* (23–25). In this study, we successfully expressed the soluble Mfa1 protein of *P. gingivalis* and generated three specific mAbs that bind with high affinity to Mfa1 through immunizing mice and hybridoma fusion technology. Importantly, these mAbs promote *P. gingivalis* agglutination, reduce its binding to sHA, and block the adhesion and invasion of gingival fibroblasts by *P. gingivalis*. Furthermore, anti-Mfa1 mAb 2D9 was found to reduce oral bacterial burden and improve periodontitis in an experimental model of periodontitis, suggesting the potential of anti-Mfa1 mAbs as anti-*P*. *gingivalis* agents for treating periodontal diseases.

*P. gingivalis* is a complex and enigmatic anaerobic pathogen, which is considered to be the key pathogenic bacteria of subgingival plaque in patients with periodontitis (26). Recently, *P. gingivalis* has been linked to systemic inflammatory diseases beyond periodontal disease, which has attracted the attention of numerous researchers (2, 3, 27). However, the lack of relevant tools, particularly antibodies, presents a major hurdle in studying the contribution of *P. gingivalis*’ virulence factors to its pathogenic properties. In this study, we developed three mAbs with different binding affinities for Mfa1. These mAbs specifically recognized both denatured and native Mfa1 protein of *P. gingivalis*, with no cross-reactivity observed with other proteins or bacteria. While PCR is a common technique for pathogen detection, it cannot be readily performed at the dental chairside (28). Immunodiagnostic methods based on mAbs offer a potential solution, enabling the rapid detection and quantification of *P. gingivalis* in associated diseases. The Mfa1 gene has been identified to have two predominant genotypes, namely 70 (kDa) and 53 (kDa), exhibiting significant differences in molecular weight, amino acid sequence, and antigenic properties (29, 30). In this study, the type 70 Mfa1 (from ATCC 33277) was used as the antigen to produce anti-Mfa1 mAbs, although it is uncertain whether it recognizes the type 53 Mfa1. However, as genotype 70 is more frequently detected in clinical samples and laboratory-preserved strains, it represents a significant target for therapeutic interventions (30, 31). These findings establish the potential application of these anti-Mfa1 mAbs as key components for the development of targeted *P. gingivalis* assays, which could facilitate oral health management and the treatment of systemic diseases.

Antibodies are crucial in combating bacterial infections by employing various mechanisms, including agglutination, neutralization, and opsonization, which impede bacterial function and enhance their elimination (22). Indeed, mAbs-mediated bacterial aggregation can provide several advantages in fighting infections, including enhanced immune recognition and reduced bacterial movement and adhesion, thus limiting the spread of infection. Specific antibody against *Streptococcus pneumoniae* induces bacterial agglutination and prevents bacterial colonization (17). Similarly, mAbs directed against *Bordetella* pertussis filamentous hemagglutinin (FHA) and fimbriae 2 and 3 (FIM) proteins induce bacterial agglutination and inhibit bacterial adhesion to epithelial cells *in vitro* (32). Recent research suggests that recombinant human secretory IgA protects against *Salmonella* Typhimurium infection by inducing bacterial agglutination, thus limiting the bacterium’s ability to invade gut-associated lymphoid tissues (33). Although no previous studies have evaluated the efficacy of mAbs in promoting *P. gingivalis* aggregation, an IgY antibody produced by immunization with lysate of membrane proteins from *Aggregatibacter actinomycetemcomitans* and *P. gingivalis* has been shown to promote agglutination of both bacteria (34). Our findings indicate that the anti-Mfa1 mAbs can induce significant *P. gingivalis* auto-aggregation, which benefits by blocking bacterial spread and modulating opsonophagocytosis (17, 33). *P. gingivalis* biofilms play a crucial role in its ability to evade host defenses and cause periodontal disease (35). Mutation of the Mfa1 protein in *P. gingivalis* leads to increased formation of single-colony biofilms, suggesting a role for Mfa1 in regulating biofilm formation. Although anti-Mfa1 antibodies promote bacterial aggregation, their effects on biofilm formation are not fully analogous to those seen with Mfa1 deletion (15, 16). Biofilm formation and microbial adhesion are two related and complex phenomena. The characteristics of the bacteria, such as their motility, surface proteins, and extracellular polysaccharides (EPS), can significantly impact biofilm formation (36). This implies that anti-Mfa1 mAbs may have additional roles in *P. gingivalis* biofilm formation, warranting further examination and discourse.

Adhesion and invasion are the initial key steps in bacterial colonization and infection. Fimbriae are proteinaceous bacterial appendages that play critical roles in biofilm formation, host cell and tissue adhesion, and bacterial interactions, making them promising vaccine antigens (37). *P. gingivalis* has two types of fimbriae, FimA and Mfa1, which play crucial roles in colonization by associating with other bacteria and host tissues (8). Both fimbriae belong to the type V class due to their polymerization through a protease-mediated donor-strand exchange mechanism (38). Knockout strains lacking FimA or Mfa1 in *P. gingivalis* exhibited reduced adhesion to human gingival epithelial cells (9). Alveolar bone loss, the hallmark of periodontitis induced by *P. gingivalis*, was significantly lower in knockout strains compared to the wild-type strain, indicating the critical roles of FimA and Mfa1 in *P. gingivalis* pathogenesis (9, 39). Mfa1 mainly mediates the heterotypic community of *P. gingivalis* and *Streptococcus* by interacting with surface antigens I/II and modulates self-aggregation and biofilm formation (40, 41). While FimA fimbriae appear to be primarily responsible for *P. gingivalis* adhesion to host cells, the role of Mfa1 in this process is less well understood. Indeed, inactivation of FimA does not entirely eliminate *P. gingivalis* adhesion to epithelial cells, suggesting that Mfa1 may contribute to adhesion through an unknown mechanism (9). Notably, the alveolar bone loss of rats infected with the FimA-deficient strain was higher than that of those infected with the Mfa1-deficient strain (8). Studies show that anti-FimA antibodies block the adhesion of *P. gingivalis* to sHA and host cells, reducing oral infection and alveolar bone loss (20, 21). In this study, we demonstrated that anti-Mfa1 mAbs significantly inhibited *P. gingivalis* adhesion to sHA, as well as to hGFs and CAL27 cells, which are representative of gingival fibroblasts and oral squamous cell carcinoma, respectively. Additionally, these antibodies significantly reduced the transcript levels of the pro-inflammatory cytokines IL-1β and IL-6, which are markers of periodontitis.

Periodontitis is a common oral inflammatory disease caused by a variety of periodontal pathogenic bacteria. The main symptoms of periodontal disease are redness, swelling, and bleeding of gum tissue, formation of periodontal pockets, and alveolar bone resorption. Effective control and removal of pathogenic bacteria in periodontal pockets is crucial for the prevention and treatment of periodontitis. Minimizing the colonization and recolonization of pathogenic microorganisms in periodontal pockets is crucial to effectively prevent and treat periodontal disease (42). Scaling and root planing in combination with systemic antibiotics is an effective clinical regimen for treating periodontitis. However, it is worth noting that scaling and root planing alone do not completely eliminate the colonized bacteria in the periodontal pockets (43), and excessive use of antibiotics can lead to increased antibiotic resistance in bacteria (44, 45). Alternative antibody-based approaches are thought to address the need for effective therapies against drug-resistant bacteria (24, 25). Antibodies targeting bacterial outer membrane proteins are a promising avenue of research, as many outer membrane proteins play key roles in adhesion, immune evasion, and bacterial biogenesis. MEDI3902, a bispecific antibody that targets *P. aeruginosa* pilus protein PcrV and the exopolysaccharide Psl, is a successful example of this strategy (46). Although Mfa1 is a critical component of *P. gingivalis* fimbriae and plays an important role in colonization and inflammation, a lack of specific antibodies has limited the evaluation of Mfa1-targeted antibody therapy. In *P. gingivalis*-infected CIA mice, FimA Ab alleviated oral disease and arthritis by disrupting *P. gingivalis* attachment to fibroblasts and inhibiting *P. gingivalis* migration to joints (20). Our findings extend these findings by demonstrating that Mfa1-targeted antibodies can reduce *P. gingivalis* burden and improve periodontitis symptoms in rats, highlighting the therapeutic potential of mAbs targeting Mfa1. Further research into the optimal dosing regimen, route of administration, and duration of treatment with anti-Mfa1 mAbs is required to assess their efficacy in treating established *P. gingivalis* infections. These studies will enhance the translational potential of our findings.

In conclusion, our study describes the development and characterization of the novel monoclonal antibodies against *P. gingivalis* Mfa1. Anti-Mfa1 mAbs have a protective effect on the periodontitis model infected by *P. gingivalis* by promoting bacterial agglutination and reducing adhesion. Future research directions include the characterization of more specific epitopes of Mfa1-mAb and investigating its contributions to biofilm formation and host immune response. Additionally, we plan to humanize the mAb through continuous transformations and evaluate its pharmacodynamics and drug performance for potential clinical applications. Overall, these results have significant implications for the development of effective diagnostic and therapeutic approaches against *P. gingivalis* infection and colonization.

## MATERIALS AND METHODS

### Bacterial strains and cells

*P. gingivalis* (ATCC33277) was inoculated onto blood agar plates and cultured at 37°C anaerobically (90% N_2_, 5% H_2_, and 5% CO_2_) for 5–7 d. Then, *P. gingivalis* strains were grown anaerobically in Brain Heart Infusion (BHI) medium supplemented with hemin (5 µg/mL) and vitamin K1 (5 µg/mL) for over 24 h at 37°C. hGFs were established from gingival tissue samples from healthy individuals as previously described (47). The process has been approved by the Ethics Committee of Henan University School of Medicine. The hGFs and CAL27 cells were cultured in Dulbecco's modified Eagle's medium (DMEM) with 10% fetal bovine serum (FBS) at 37°C and 5% CO_2_.

### Production and purification of recombinant Mfa1 proteins

The full-length Mfa1 was amplified with primers by PCR from the reference strains *P. gingivalis* ATCC-33277 genome. The recombinant plasmid (pET-28a-Mfa1) was constructed as a C-terminal 6× His-tagged from plasmid pET-28a (+). Plasmids were transformed into *E. coli* BL21 (DE3) and grown in Luria Bertani (LB) media supplemented with 50 µg/mL kanamycin at 37°C. The bacterial solution was amplified and expression induced, followed by centrifugation after ultrasonic lysis. The antigen was purified with a Ni-NTA resin purification kit, and the solvent was replaced with phosphate-buffered saline (PBS). Various elution fractions were collected and analyzed by SDS-PAGE and Western blot to verify purity.

### Generation of mAbs against Mfa1 of *P. gingivalis*

All animal care and experimental procedures were approved by the Animal Ethics Committee of Henan University (Approval No. DWLL20220308). BALB/c mice (6–8 weeks, Vital River, Beijing) were immunized subcutaneously with recombinant Mfa1 mixed with Freund’s adjuvant every 2 wk. Antigen-reactive hybridoma cells were obtained through fusion of splenocytes and SP2/0 cells and then expanded. BALB/c mice were sensitized with autoclaved paraffin, and the hybridoma cells were injected intraperitoneally. The ascites fluid was collected after 7 d, and the antibodies were purified using Protein A/G affinity chromatography.

### Enzyme-linked immunosorbent assay (ELISA)

The recombinant Mfa1 protein was coated onto 96-well ELISA plates at a concentration of 2 µg/well and was incubated overnight at 4°C. The plates were then blocked with 5% skim milk and incubated with cell supernatants or mAbs (including non-specific IgG), followed by a horseradish peroxidase (HRP)-labeled goat anti-mouse IgG secondary antibody, which was used for signal development. Data were plotted through GraphPad Prism, and nonlinear regression analysis was performed to calculate the half-maximal binding concentration. To detect the binding of anti-Mfa1 mAbs to *P. gingivalis* and other bacteria, bacterial cells were washed in PBS and diluted in PBS to an optical density of 0.5 and coated onto a 96-well ELISA plate, with the addition of 1% bovine serum albumin (BSA) to reduce non-specific binding.

### SDS–PAGE and Western blotting

Protein samples were treated with SDS extraction buffer and were loaded into SDS-PAGE gel. The gel was stained with Coomassie Brilliant Blue G250. For Western blotting, proteins were transferred onto polyvinylidene fluoride (PVDF) membranes (48). Membranes were blocked with 5% skim milk at room temperature for 1 h to block non-specific binding. The membranes were incubated with purified anti-Mfa1 mAbs (4°C, overnight), followed by incubation with the corresponding by HRP-conjugated secondary antibody (Sigma, USA) for 1 h at room temperature. Then, the membranes were detected and visualized using ECL detection reagents (Thermo, USA).

### Bio-layer interferometry (BLI) kinetics assay

Binding kinetics of Mfa1-mAb were performed using an Anti-mouse IgG Fc Capture (AMC) sensor (Sartorius, Germany) in an Octet R8 instrument (Sartorius, Germany). The sensors were dipped into baseline 1× Octet Kinetics Buffer and incubated with anti-Mfa1 mAbs (1 µg/mL) as a loading step. Then, the sensors were immersed in a second baseline containing 1× Octet Kinetics Buffer. Sensors were then immersed in varying nanomolar concentrations of recombinant Mfa1 protein (from 6.25 to 200 nM) as an association step and then transferred back to 1× Octet Kinetics Buffer as a dissociation step. KD was determined from the BLI curve obtained from Mfa1 concentration analysis (Koff/Kon). Data from binding kinetics curves were fit globally using a 1:1 binding model (Octet Analysis Studio v13.0, Sartorius).

### Immunofluorescence staining

To detect surface binding of purified anti-Mfa1 mAbs to *P. gingivalis*, cells were stained with Hochest’s reagent at 37°C for 15 min and washed. *P. gingivalis* was incubated with anti-Mfa1 mAbs or non-specific IgG at 37°C for 1 h, followed by incubation with Alexa Fluor 546-conjugated anti-mIgG secondary antibody (Thermo, USA) at 37°C for 30 min. Then, the *P. gingivalis* was resuspended in 50 µL of PBS and dropped on slides. Images were analyzed using a fluorescence microscope (Zeiss, AxioImager A2, USA).

### Flow cytometry assay

To assess antibody binding to the *P. gingivalis* surface Mfa1 protein, *P. gingivalis* was washed and resuspended in PBS supplemented with 0.1% NaN_3_ and 2% BSA. Anti-Mfa1 mAbs or non-specific IgG was added to the *P. gingivalis* suspension, mixed gently, and incubated for 1 h at room temperature. Cells were washed and incubated with Alexa Fluor 488-conjugated anti-mIgG secondary antibody (Thermo, USA) at 37°C for 30 min. After washing three times, bacteria were resuspended in 1× PBS and transferred to a 5-mL flow tube. Flow cytometric analysis was performed on a ZE5 Cell Analyzer (Bio-Rad, USA), analyzed using FlowJo software (BD Biosciences, USA).

### Quantitative PCR

The total genome DNA of *P. gingivalis* was isolated using a DNA Isolation Mini Kit (Vazyme, China) according to the manufacturer’s instructions, and quality was assessed using a Nanodrop 2000 (Thermo, USA). The *P. gingivalis*-specific primers and probes were designed based on species-specific conserved regions of the 16S rRNA gene (28). To create a quantitative analysis, the optical density of the bacterial suspension was measured at 600 nm using a spectrophotometer, with a value of 1 corresponding to 1 × 10^9^ CFU/mL. DNA extracted from *P. gingivalis* was used to prepare a standard curve and served as a positive control, while sterile water was used as a negative control. The quantitative real-time PCR of IL-1β and IL-6 and the primers were previously described (48). Briefly, total RNA was extracted from cells using TRIzol reagent (TaKaRa, Japan). cDNA was synthesized by reverse transcription of 1 µg total RNA using a SYBR qPCR SuperMix (Vazyme, China). qPCR was performed using a 7500Fast PCR system (ABI, USA).

### Antibacterial and auto-agglutination assays

To evaluate the antibacterial effect of Mfa1-mAb against *P. gingivalis*, plate colony CFU count detection was performed. *P. gingivalis* was washed and resuspended in PBS (1 × 108 CFU/mL), and incubated with anti-Mfa1 mAbs (100 μg/mL) and/or non-specific IgG at 37°C for 2 h. The mixture was briefly shaken, and then 10-fold serial dilutions of the different groups of bacteria were spread on blood agar plates. After being cultured at 37°C for 7–10 d under anaerobic conditions, the plates were scanned to count bacterial colonies. The average CFUs were adjusted for dilution factors to determine the CFU per sample. In order to provide a more comprehensive analysis of the decrease in the number of *P. gingivalis* clones following incubation with anti-Mfa1 mAbs, the LIVE/DEAD backlight bacteria assay was performed. Briefly, 1 × 10^7^ *P. gingivalis* cells were incubated with 100 µg/mL of anti-Mfa1 mAbs or IgG for 2 h at room temperature. The incubated mixture was stained with the LIVE/DEAD Backlit Bacterial Viability Kit containing DMAO (green for bacterial live) and EthD-III (red for bacterial death) for 15 min. The mixture was washed twice with PBS and briefly vortexed. Subsequently, fluorescence photographs were taken using a fluorescence microscope (Axio-Imager A2, Zeiss, USA), and flow cytometry analysis was conducted on a ZE5 Cell Analyzer (Bio-Rad, USA).

The microbial agglutination assay was performed based on the protocol published earlier (49). Briefly, *P. gingivalis* was washed and resuspended in PBS. In a UV transparent cuvette, *P. gingivalis* was incubated with anti-Mfa1 mAbs and non-specific IgG, and statically maintained at 37°C. Absorbance at 600 nm was continuously read for 2.5 h. To visualize and quantify bacterial agglutination, the bacterial concentration was adjusted to 2 × 10^7^ CFU/mL in PBS, and then incubated with different concentrations of anti-Mfa1 mAbs and non-specific IgG. Drops (10 µL) of the sample were placed onto glass slides, covered with a coverslip, and sealed with a nail polish. The samples were then observed using a fluorescence microscope (Axio-Imager A2, Zeiss, USA). For aggregation analysis, 20 randomly imaged areas were analyzed for the number and size of aggregates using ImageJ.

### Binding to saliva-coated hydroxyapatite (sHA) bead assay

To test whether Mfa1-mAb has the ability to block *P. gingivalis* adhesion, an assay of the ability of *P. gingivalis* to bind sHA beads was performed as previously described (50). Briefly, human whole saliva-coated hyaluronic acid (sHA) beads were prepared by incubating sHA beads with clarified human whole saliva *in silica*-coated borosilicate tubes overnight. The sHA beads and *P. gingivalis* were washed twice with KCl buffer. Next, a mixture of *P. gingivalis*, sHA beads, and anti-Mfa1 mAbs or non-specific IgG was prepared and incubated under gentle oscillation at room temperature for 1 h. After incubation, the mixture was layered on 1 mL of Percoll (Solarbio, China). sHA beads with bound *P. gingivalis* were washed once with Percoll and twice with KCl buffer. The mixture was then used for genomic DNA extraction, and the amount of *P. gingivalis* bound to the sHA beads was evaluated by qPCR. For visualization by SEM, the sHA beads with bound *P. gingivalis* were immersed in a fixative solution (5% glutaraldehyde, 0.1 M phosphate, pH 7.0) for 12 h at 4℃. Samples were rinsed with 0.1 M phosphate buffer (PB), dehydrated in a graded ethanol series, and dried using a critical point dryer (EM CPD300, Leica, Germany). lmages were captured using a field emission scanning electron microscope (ESEM Prisma E, Thermo, USA).

### Biofilm assays

*P. gingivalis* biofilm formation was quantified in a 24-well polystyrene cell culture dish using a microtiter plate assay (34). Briefly, 1 × 10^8^ *P. gingivalis* suspension was inoculated into the wells of a flat-bottomed 24-well polystyrene plate, followed by the addition of 100 µg of different anti-Mfa1 monoclonal antibodies and non-specific IgG, respectively. After incubation under anaerobic conditions for 48–72 h, the planktonic bacterial cells in the wells were discarded, washed three times with PBS, air dried, and then stained with 500 µL of 0.5% (wt/vol) crystal violet for 15 min. Excess dye was eluted with sterile water, and the cell-bound dye was eluted using 500 µL of absolute ethanol. Biofilm formation was quantified by measuring the absorbance at a wavelength of 595 nm. For SEM observation of biofilm formation, glass cell slides were first placed at the bottom of the cell culture dish. Suspension of 1 × 10^8^ *P. gingivalis* cells was then inoculated into each well. One hundred micrograms of different anti-Mfa1 monoclonal antibodies and non-specific IgG was added to the wells, respectively, and the cultures were incubated under anaerobic conditions for 48–72 h. After discarding the planktonic bacterial cells, the biofilm-covered cell slides were gently washed three times with PBS. After fixation, the biofilm-coated cell slides were rinsed and dehydrated in ethanol, dried, and then gold sprayed. The slides were then visualized and photographed using emission scanning electron microscopy (ESEM Prisma E, Thermo, USA).

### Detection of adhesion and invasion of epithelial cells by *P. gingivalis*

To evaluate whether anti-Mfa1 mAbs have an effect in blocking the adhesion and invasion of host cells by *P. gingivalis*, hGFs and CAL27 cells were cultured in monolayers and exposed to *P. gingivalis*. Briefly, approximately 1 × 10^6^ cells per well were seeded in 6-well plates overnight and washed twice with additive-free DMEM. The cells were infected at a multiplicity of infection (MOI = 10) for 1 h at 37°C with rocking every 15 min, anti-Mfa1 mAbs were added simultaneously, and non-specific IgG was used as a negative control. Unbound *P. gingivalis* was washed away, and the cells were continued in complete medium for 4 h. Next, they were used for qPCR detection. Immunofluorescence staining analysis was performed to visualize the adhesion and invasion effects of *P. gingivalis* on hGFs and CAL27 cells. *P. gingivalis* was stained by FITC before infection. Cells were stained with Fluorescein Phalloidin for F-actin and DAPI (4′,6-diamidino-2-phenylindole) for nuclear DNA. Images were analyzed using a fluorescence microscope (AxioImager-A2, Zeiss, USA) or a confocal laser-scanning microscope (A1R, Nikon, USA).

Furthermore, we employed the well-established antibiotic protection assay to accurately assess the invasion of epithelial cells by *P. gingivalis*. Briefly, 1 × 10^5^ hGFs cells or 1 × 10^6^ CAL27 cells were cultured overnight in 12-well plates, washed twice with DMEM. The cells were infected at a multiplicity of infection (MOI = 100) for 1 h at 37°C, anti-Mfa1 mAbs were added simultaneously, and non-specific IgG was used as a negative control. The cells were washed with PBS to remove non-adherent bacteria and treated with 200 µg/mL metronidazole in DMEM for 1 h at 37°C to eliminate adherent external bacteria. The cells were then washed three times with PBS and lysed with distilled water for 30 min while mechanical scraping was performed to release the bacteria inside. The lysate was diluted 100-fold, seeded on blood agar plates, and incubated under anaerobic conditions at 37°C for 7 d. Viable cells were then counted and analyzed.

### Establishment and assessment of experimental periodontitis

To assess the blocking effect of Mfa1-mAb on *P. gingivalis*-induced periodontitis in SD rats (160–180 g, Vital River, Beijing), a rat model of periodontitis was established through ligation and bacterial inoculation. Rats were anesthetized with sodium pentobarbital and a piece of sterile cotton thread ligated around the upper left first molar. To evaluate the efficacy of Mfa1-mAb in a *P. gingivalis*-induced periodontitis model, SD rats were randomly divided into five groups (normal group, untreated group, *P. gingivalis* group, non-specific IgG group, and mAb group), and the rat periodontitis model was induced by ligation and *P. gingivalis* inoculation. Rats were lightly anesthetized with sodium pentobarbital, and a cotton ligature was placed around the maxillary second molars on both sides of rats in the untreated group, *P. gingivalis* group, non-specific IgG group, and mAb group. Rats were orally inoculated with *P. gingivalis* (2 × 10^9^ CFU), which was suspended in 100 µL of PBS containing 2% carboxymethyl cellulose, every 2 d for 15 d. In the mAb group, *P. gingivalis* was incubated with anti-Mfa1 mAb (200 µg/mL) and resuspended in 50 µL of PBS with 2% carboxymethyl cellulose, followed by inoculation of rats.

To assess the bacterial burden of *P. gingivalis* in the oral cavity, a sterile swab was used to collect oral flora 1 d after the final *P. gingivalis* injection. Bacterial burden was assessed by qPCR analysis of the *P. gingivalis* 16S gene. The mandibles were fixed with paraformaldehyde to observe the alveolar bone loss in different groups and to assess the effect of treatment on bone tissue. Three-dimensional images of the buccal and palatal sides were reconstructed with a Micro-CT (Quantum GX2, PE, USA) and were analyzed using the Radiant Dicom Viewer image analysis system. Three-dimensional images of the buccal and palatal sides were reconstructed with a Micro-CT (Quantum GX2, PE, USA), and an image analysis system Radiant Dicom Viewer. The distance from the CEJ to the ABC was measured around the second molars. The three-dimensional images were used to analyze bone volume/total volume (BV/TV). To observe periodontal tissue inflammation, the mandible was fixed with 10% paraformaldehyde, decalcified, and embedded in paraffin. The tissue blocks were made into tissue sections through buccal and lingual direction, which were stained with H&E and observed under a light microscope.

### Statistical analysis

Data were representative of three or more independent experiments, and all results were expressed as mean values  ±  standard error of the mean. Statistical analysis was performed using Graphpad Prism software (San Diego, CA, USA). Quantitative data were evaluated by one-way analysis of variance (ANOVA); *P < 0.05 and **P < 0.01 were considered statistically significant.

## SUPPLEMENTAL MATERIAL

Figure S1 msphere.00721-24-s0001.docx Purification and characterization of anti-Mfa1 mAbs.

ASM does not own the copyrights to Supplemental Material that may be linked to, or accessed through, an article. The authors have granted ASM a non-exclusive, world-wide license to publish the Supplemental Material files. Please contact the corresponding author directly for reuse.
