# Supplementary material for: Development of monoclonal antibodies against P. gingivalis Mfa1 and their protective capacity in an experimental periodontitis model
Source: mSphere. 2024 Dec 19;10(1):e00721-24. doi: 10.1128/msphere.00721-24 (PMC11774036; doi:10.1128/msphere.00721-24)
Supplement: Figure S1 — Purification and characterization of anti-Mfa1 mAbs. [file msphere.00721-24-s0001.docx]

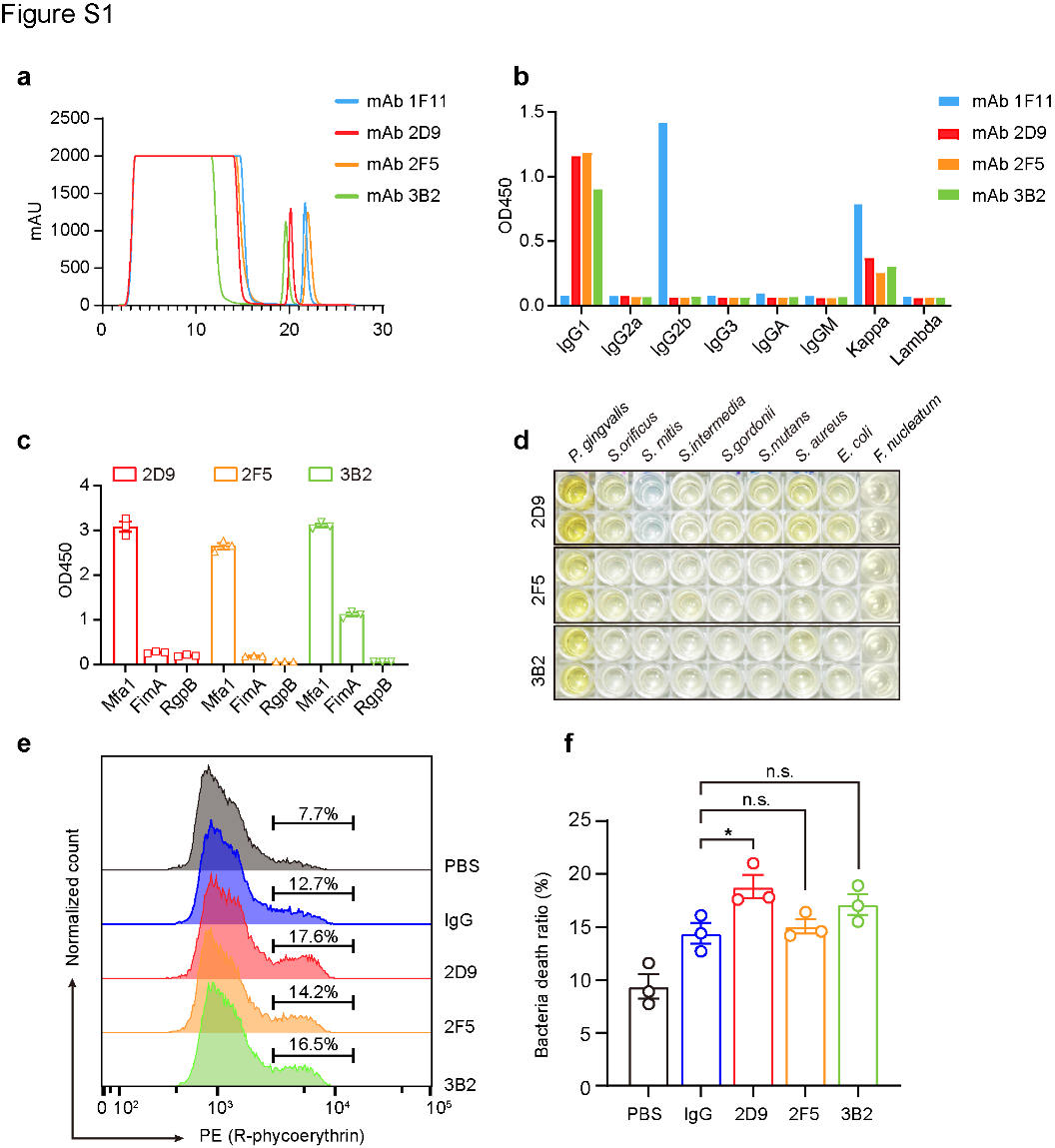


**Supplementary Figure 1 Purification and characterization of anti-Mfa1 mAbs.** (a) Four anti-Mfa1 monoclonal antibodies (mAbs) were purified from ascites fluid using protein A/G affinity chromatography via the AKTA protein purification system. (b) Isotypes of anti-Mfa1 mAbs were characterized by ELISA. (c) The specificity of the four anti-Mfa1 mAbs (mAbs) was assessed by ELISA using different antigens of *P. gingivalis* coated onto multi-well plates. (d) Cross-reactivity of the mAbs with other pathogenic bacteria was determined using ELISA, and end-point images were acquired to visualize the results. (e) Bacterial flow cytometry analysis was performed by incubating *P. gingivalis* with anti-Mfa1 mAbs and IgG for 2 hours, and stained with EthD-III. (f) The histogram depicts the percentage of EthD-III positive cells (which represent dead cells) following each treatment. Mean and s.e.m. were calculated from results of at least three independent experiments. **p*<0.05 as determined by a one-way ANOVA analysis.
